# Prevalence of Slowly Expanding Lesions in Patients With Multiple Sclerosis: A Systematic Review and Meta-Analysis

**DOI:** 10.1212/WNL.0000000000218474

**Published:** 2026-09-01

**Authors:** Andreas Liampas, Artemios Artemiadis, Vasilis-Spyridon Tseriotis, Ferran Prados, Paolo Preziosa, Alberto Calvi, Georgios Hadjigeorgiou, Maria A. Rocca, Massimo Filippi, Olga Ciccarelli

**Affiliations:** 1 Queen Square MS Centre, Department of Neuroinflammation, UCL Queen Square Institute of Neurology, Faculty of Brain Sciences, University College London, United Kingdom;; 2 Department of Neurology, Medical School, University of Cyprus, Nicosia;; 3 Department of Neurology, Agios Pavlos General Hospital of Thessaloniki, Greece;; 4 Laboratory of Clinical Pharmacology, Aristotle University Campus, Aristotle University of Thessaloniki, Greece;; 5 Hawkes Institute, Department of Medical Physics and Biomedical Engineering, University College London, United Kingdom;; 6 Universitat Oberta de Catalunya, Barcelona, Spain;; 7 Neuroimaging Research Unit, Division of Neuroscience, IRCCS San Raffaele Scientific Institute, Milan, Italy;; 8 Neurology Unit, IRCCS San Raffaele Scientific Institute, Milan, Italy;; 9 Vita-Salute San Raffaele University, Milan, Italy;; 10 Neuroimmunology and Multiple Sclerosis Unit and Laboratory of Advanced Imaging in Multiple Sclerosis (ImaginEM), Hospital Clinic Barcelona, Spain;; 11 Neurorehabilitation Unit, IRCCS San Raffaele Scientific Institute, Milan, Italy;; 12 Neurophysiology Service, IRCCS San Raffaele Scientific Institute, Milan, Italy; and; 13 National Institute for Health and Care Research (NIHR), University College London Hospitals (UCLH) Biomedical Research Centre, United Kingdom.

## Abstract

**Background and Objectives:**

Chronic active lesions (CALs) reflect chronic inflammation in multiple sclerosis (MS). Slowly expanding lesions (SELs) are CALs identified on conventional MRI by linear, concentric expansion over time, while paramagnetic rim lesions (PRLs) are CALs characterized by a paramagnetic rim on susceptibility-sensitive MRI. However, the prevalence of SELs and their overlap with PRLs remain unclear. The aims of this study were to (1) estimate the proportion of SELs among all T2 lesions and the proportion of patients with at least 1 SEL and (2) assess the proportion of SELs overlapping with PRLs.

**Methods:**

We systematically searched PubMed, Scopus, Web of Science, and Embase on February 1, 2026, for studies evaluating SELs in MS. At least 2 authors independently assessed study eligibility. Primary outcomes were the pooled proportion of SELs among T2 lesions and the proportion of patients with at least 1 SEL. We estimated mean per-patient volumes of SELs and total T2 lesions and the proportion of SELs overlapping with PRLs. Random-effects generalized linear mixed-effects models and inverse-variance methods were used, with between-study heterogeneity assessed using τ^2^ and *I*^2^ and robustness using sensitivity analyses. Univariable meta-regression explored heterogeneity. PROSPERO: CRD42024603778.

**Results:**

Of 5,980 records, 20 studies comprising 4,786 patients with MS were included (mean age: 43.6 ± 6.3 years; 63.7% female). Sample sizes varied by outcome. SELs accounted for 14% (95% CI 10–21) of all T2 lesions, and 78% (67–85) of patients had at least 1 SEL. After sensitivity analysis, per-patient mean volumes were 1.42 mL (0.79–2.06) for SELs and 10.6 mL (8.53–12.67) for total T2 lesions. In total, 11% (6–20) of SELs overlapped with PRLs. In subgroup analyses, proportions of SELs were similar in relapsing-remitting and progressive MS (15%), but the proportion of patients with at least 1 SEL was higher in progressive MS. Between-study heterogeneity was high across analyses with no significant sources identified.

**Discussion:**

Although SELs represent a minority of T2 lesions, most patients have at least 1 SEL and a subset overlaps with PRLs, suggesting a partial correspondence between these 2 imaging markers of chronic inflammatory activity. Limitations include possible publication bias, high unexplained heterogeneity, differences in SEL identification methods, and differences in MRI time point number/timing.

## Introduction

Multiple sclerosis (MS) is a chronic immune-mediated disease of the CNS characterized by demyelinating lesions that drive neurodegeneration and progressive neurologic disability. Pathologic studies have demonstrated that chronic active lesions (CALs) constitute a substantial proportion of MS pathology, accounting for up to 57% of all lesions,^1^ with a higher prevalence in secondary progressive MS (SPMS) compared with relapsing-remitting MS (RRMS).^1^ Imaging biomarkers of CALs are slowly expanding lesions (SELs) and paramagnetic rim lesions (PRLs).

SELs are characterized by concentric and linear expansion on serial T1-weighted and/or T2-weighted scans (which are conventional MRI scans) using nonrigid, longitudinal, deformation-based analysis.^2^ SELs are observed in all MS phenotypes,^2^ although more prominent in progressive MS^3^ and predict disability progression^4^; they may represent an underlying mechanism of progression independent of relapse activity (PIRA).^5^ Although lacking a complete histopathologic validation,^6^ a recent immunologic and molecular profiling study reported that SELs are characterized by a hypocellular core and a rim of macrophages/microglia at the lesion edge, where ongoing myelin degradation and axonal damage occur.^7^ Resident microglia with M1 (proinflammatory phenotype) differentiation were detected at the lesion edge.^7^ Gene expression profiling of SEL borders further revealed upregulation of immune-related and inflammation-related pathways, including genes involved in microglial activation, lipid metabolism, and neurodegeneration, supporting a central role for microglia-driven chronic inflammation in lesion expansion.^7^ Current studies show considerable variability in SEL detection methods and reported prevalence.

PRLs are detected on susceptibility-sensitive MRI; they show a paramagnetic rim, which reflects iron-laden activated microglia/macrophages at the lesion edge, and a T2-hyperintense core, which does not enhance with gadolinium contrast agent.^2^ One study has shown that SELs are much more numerous than PRLs, and only a minority of SELs is known to have PRL features.^8^ The proportion of overlap between SELs and PRLs across studies is unknown.

Given the growing interest in SELs as biomarkers of chronic active inflammation and their potential clinical applicability, robust pooled estimates are needed to better define their burden across MS populations and inform their use in clinical and research settings. The aims of this study were to establish the pooled proportion of SELs out of all T2 lesions in people with MS and the proportion of patients with at least 1 SEL, together with the pooled per-patient mean volumes of SELs. As a secondary aim, we investigated the pooled proportion of SELs that overlap with PRLs.

## Methods

### Standard Protocol Approvals, Registrations, and Patient Consents

This systematic review with meta-analysis was conducted based on our published protocol (PROSPERO CRD42024603778) and reported according to the Preferred Reporting Items for Systematic Reviews and Meta-Analyses (PRISMA) statement^9^ (eAppendix 1 and 2) and the Meta-analysis of Observational Studies in Epidemiology guidelines^10^ (eAppendix 3). Deviations from the original PROSPERO-registered protocol are detailed in eAppendix 4.

### Search Strategy

We conducted a systematic review of all peer-reviewed, published studies reporting the prevalence of SELs in people with MS published before February 2, 2026, in 4 databases, including PubMed, Scopus, Web of Science, and Embase (eAppendix 5). In PubMed and Scopus, searches were restricted to studies published in English and involving human subjects. Furthermore, the reference lists of all included studies were manually screened to identify further eligible publications.

### Selection Criteria

First, all records were automatically deduplicated using Rayyan.^11^ Titles and abstracts were then screened on Rayyan independently by 2 reviewers (A.L. and V.S.T.), blinded to each other's decisions. Abstracts not meeting the inclusion criteria were excluded, and disagreements were resolved by consensus with a third reviewer (A.A.). Full-text screening was subsequently performed independently by the same reviewers, with conflicts resolved using the same procedure. To identify potential duplicate reporting of cohorts, corresponding authors were contacted when necessary. Studies were eligible for inclusion if they met the following criteria: (1) included adult patients (aged 18 years or older) with MS according to the McDonald criteria (any version), (2) involved MRI acquisitions including T1-weighted and/or T2-weighted sequences and fluid-attenuated inversion recovery (FLAIR) sequences, (3) reported data on the number and/or volume of SELs, and (4) were written in English. No restrictions were applied with respect to geographic region or clinical setting. Manuscripts were excluded if they (1) were review articles, letters, editorials, opinions, or conference abstracts; (2) were based on postmortem findings or imaging techniques other than conventional MRI; and (3) involved preclinical models. When multiple publications reported data from the same cohort, all relevant prevalence estimates were considered; however, only 1 study per cohort was included in each meta-analysis. For 2 overlapping cohorts one study was included in lesion-level and mean volume analyses,^13 ^while the other was included in patient-level meta-analysis,^12^ according to data availability (following direct correspondence with the authors). When required data were missing, corresponding authors were contacted (with 1 follow-up); studies were excluded if data remained unavailable.

### Data Extraction

Data were extracted from each study using a structured coding scheme in Excel, including the following details: study characteristics (design and study time frame); patients' cohorts characteristics (sample size, sex, mean age, MS phenotype, disease duration, baseline Expanded Disability Status Scale [EDSS] scores, duration of follow-up, and disease-modifying therapy [DMT] exposure); MRI scan characteristics (scanner field strength and scanner type); number and volume of SELs and total T2 lesions; methodological information related to SEL identification, including imaging sequences used for primary SEL detection and lesion masking; the minimum expansion threshold applied, concentricity, and constancy criteria; and the time frame of SEL identification. Disagreements were resolved by discussion or adjudication by a third reviewer (A.A.). Authors of included studies were contacted for clarification or additional data if necessary.

### Quality Assessment

Methodological quality of all articles was independently evaluated by 2 reviewers (A.L., A.C.) using the Joanna Briggs Institute Critical Appraisal Checklist for Studies Reporting Prevalence Data,^13^ which assesses key domains including sample representativeness, sampling methods, adequacy of sample size, validity and reliability of outcome measurement, completeness of data, and appropriateness of statistical analysis. A total quality score from 0 to 9 was calculated for each study (eAppendix 6). Although some cohorts originated from randomized controlled trials (RCTs), SEL outcomes were derived from post hoc imaging analyses; therefore, these cohorts were appraised as observational studies for prevalence estimation.

### Data Synthesis and Statistical Analysis

The primary outcomes of this study were the pooled proportion of SELs out of all T2 lesions, the proportion of patients with at least 1 SEL, and the pooled per-patient mean volumes of SELs and total T2 lesions. Descriptive statistics, including means and standard deviations, were calculated using automated statistical processing tools to standardize data extraction and ensure methodological consistency across studies.^14^ R version 4.5.0 was used for the quantitative synthesis of our results.^15^ We built a generalized linear mixed-effects model^16,17^ for proportional meta-analyses with the employment of the “meta” package, fitting a logistic regression model but also including random-effects, because of the expected between-study heterogeneity. Before pooling, proportions were transformed using the Freeman-Tukey double arcsine method to stabilize variance.^17^ To synthesize continuous variables, we conducted random-effects meta-analyses of continuous outcomes using untransformed (raw) mean values to estimate pooled lesion volumes of SELs and T2 lesions from individual study arms. Pooled estimates were calculated using the inverse variance method. The restricted maximum likelihood approach was used as an estimator for between-study variance and Hartung-Knapp adjustment for CIs. Between-study variance was quantified using tau-squared (τ^2^) as an absolute measure of heterogeneity. Between-study heterogeneity was further assessed using the *I*^2^ statistic^18^ (<40%: low, >75%: significant heterogeneity) and Cochran *Q* test.^18^ Meta-analysis results were presented in forest plots showing pooled proportions and their corresponding 95% CIs, and publication bias was assessed using funnel plots. Influence and outlier assessments were performed using sensitivity analyses to evaluate the robustness of pooled estimates. For analyses of mean lesion volumes and the pooled proportion of SELs also identified as PRLs, sensitivity analyses were conducted by excluding the study with the widest CI. Pooled estimates and heterogeneity metrics were recalculated, whereas the primary analyses including all studies were retained and reported. These analyses were performed solely to assess robustness and not to exclude studies from the primary analyses.

To explore potential sources of between-study heterogeneity, we performed subgroup analyses based on MS phenotype, SEL identification methodology and study design, as well as exploratory univariable meta-regression analyses within the same random-effects framework. Lesion-level SEL prevalence was modelled on the logit scale, using the number of SELs relative to total number of T2 lesions per study. Study-level covariates were examined individually as moderators, including time frame used for SEL identification, mean age, disease duration, baseline EDSS, proportion of patients receiving any DMT, proportion receiving high-efficacy DMT, proportion receiving non–high-efficacy DMT, proportion receiving no DMT, and MRI scanner field strength. All meta-regression analyses were conducted on an exploratory basis and were not intended to adjust pooled prevalence estimates. Although detailed imaging variables were extracted, these were not included in subgroup or meta-regression analyses because of heterogeneity in definitions and reporting, as well as the limited number of studies.

### Data Availability

This study synthesizes previously published data and does not involve the collection of original human or animal data. Additional data are provided in the Supplementary Material.

### Use of AI

ChatGPT 5.0 (OpenAI, 2026) was used to assist with language refinement and clarity describing the meta-regression analysis (eMethods, eAppendix 7) and to create the comparison of the initial and final PROSEPRO protocols (eAppendix 4). All outputs were reviewed and verified by the authors, who take full responsibility for the content.

## Results

### Search Results

A total of 5,980 records were identified through the initial database search. After deleting the duplications, 3,026 unique studies remained. After title and abstract screening, 173 full-text articles were assessed for eligibility, of which 153 were excluded (reasons for exclusion were not mutually exclusive). Ultimately, 20 studies, published between 2016 and 2025, met all inclusion criteria^3,4,8,12,19-34^ and were included in the final analysis (eAppendix 8). eFigure 1 summarizes the screening process using a PRISMA flow diagram^35^ (eAppendix 9).

### Studies' and Patients' Characteristics

Twenty studies, including 4,786 patients, were included in the meta-analysis: 5 were observational prospective cohort studies,^4,20,21,24,34^ 8 were observational retrospective cohort studies,^8,12,19,26,27,29,31,32^ and 7 were secondary analyses of RCTs.^3,22,23,25,28,30,33^ Although a proportion derived from RCT data sets, SEL outcomes were obtained from post hoc imaging analyses and were therefore treated as observational for prevalence estimation. Study sample sizes ranged from 15 to 1,889 patients. Baseline MS phenotypes were RRMS (number of patients [N] = 2,514), SPMS (N = 1,439), primary progressive MS (PPMS) (N = 673), progressive-relapsing MS (PRMS) (N = 1), and clinically isolated syndrome (CIS) (N = 8). One study did not specify the MS phenotype.^29^ The mean proportion of female participants was 63.7% (range 52.9%–80%). The mean age and disease duration across studies ranged from 35.5 to 55.9 years and 4.4 to 20 years, respectively, and the mean follow-up period was 3.2 ± 2 years, although these variables were incompletely reported across studies. The mean EDSS score varied across studies (0–6.0), reflecting variability in clinical severity. MRI field strengths included 1.5T, 3T, and 7T. SELs were most commonly located in deep white matter and periventricular regions, followed by juxtacortical areas and rarer cortical and deep gray matter involvement.^3,20,22,26^ Across studies, SEL identification relied on longitudinal MRI assessments using at least 2 serial time points; however, the number and timing of imaging assessments varied, ranging from 2 time points (i.e., baseline and a single follow-up scan)^8,12,19,20,24,26,27,34^ to multiple, repeated assessments over extended follow-ups.^21-23,25,28,30-33^ Fixed imaging schedules were used in most studies,^12,19-23,25,28,30-34^ whereas others reported variable^8,24,26,27^ or incompletely specified imaging intervals.^29^ Detailed study and patient characteristics are summarized in Table 1, while MRI acquisition and SEL identification techniques are summarized in Table 2.

**Table 1:** Studies' and Patients' Characteristics

Study	Study design	SC/MC	Time frame	Sample size	MS phenotype at baseline	Age, y, mean ± SD	Sex	Disease duration, y, mean ± SD	Time frame for SEL identification, wk	Follow-up, y, mean ± SD	EDSS at baseline, mean ± SD	Type of DMT used
Fox et al. (2016)^20^	Prospective observational cohort study	SC	Not specified	92	2 CIS 69 RRMS 16 SPMS 4 PPMS 1 PRMS	54.5 ± 8.7^c^	67 F 25 M	12 ± 6.9	Baseline, 48	1	3 ± 1.5	HE-DMT: 4 NHE-DMT: 61 No DMT: 23
Absinta et al. (2019)^21^	Prospective observational cohort study	SC	2012–2018	23^a^	23 RRMS	54 ± 10	13 F 10 M	20.0 ± 7.0	Baseline, and yearly for at least 10 y	14 ± 5	Not specified	Not specified
Elliott et al. (2018)^22^	Retrospective analysis of RCT	MC	Not specified	1889	OPERA I, II: 1334 RRMS ORATORIO: 555 PPMS	OPERA I, II: 37.3 ± 9.2 ORATORIO: 44.9 ± 7.9	1149 F 740 M	OPERA I, II: 6.5 ± 6.1 ORATORIO: 6.4 ± 3.7	OPERA I, II: baseline, 24, 48 ORATORIO: baseline, 24, 48	Not specified	OPERA I, II: 2.7 ± 1.3 ORATORIO: 4.6 ± 1.2^b^	HE-DMT: 1309 NHE-DMT: 835 Placebo: 244
Elliott et al. (2020)^23^	Retrospective analysis of RCT	MC	2013–2016	299	242 RRMS 57 SPMS	39.3 ± 3^c^	196 F 103 M	Not specified	Baseline, 4, 8, 12, 16, 20, 24, 48, 72	1.5	Not specified	Not specified
Klistorner et al. (2020)^19^	Retrospective observational cohort study	MC	Not specified	33	33 RRMS	42.6 ± 10.3	20 F 13 M	6.5 ± 2.8	Baseline, 48	5	1.1 ± 1	HE-DMT: 15 NHE-DMT: 17 No DMT: 1
Klistorner et al. (2021)^12^	Retrospective observational cohort study	MC	Not specified	34	34 RRMS	42.6 ± 9.8	18 F 12 M	6.1 ± 2.9	12 (termed “baseline”), 60	5	1 ± 1	HE-DMT: 17 NHE-DMT: 15 No DMT: 2
Moog et al. (2022)^24^	Prospective observational cohort study	SC	Not specified	35	35 RRMS	40.1 ± 9.3^c^	22 F 7 M	9.4 ± 5.3^c^	Baseline, ≥24	2 ± 0.7^c^	0 ± 0^c^	HE-DMT: 14 NHE-DMT: 18
Preziosa et al. (2022)^4^	Prospective longitudinal cohort study	SC	2011–2016	52	52 RRMS	36.8 ± 9.7	30 F 22 M	9.8 ± 6.5	Baseline, 24, 48, 96	8.7 ± 1.4^c^	2.3 ± 2.9^c^	HE-DMT: 52
Beynon et al. (2022)^25^	Retrospective analysis of RCT	MC	2011–2016	887	887 SPMS	47.3 ± 7.6	550 F 337 M	Not specified	Baseline, 24, 48, 72, 96, 108	2	Not specified	HE-DMT: 439 Placebo: 448
Weber et al. (2022)^26^	Retrospective observational cohort study	SC	Not specified	43	36 RRMS 7 SPMS	40.8 ± 11.2^c^	33 F 10 M	Not specified	Baseline, ≥48	4	2.8 ± 1.4^c^	Baseline: HE-DMT: 13 NHE-DMT 17 No DMT: 13 Last Follow-up: HE-DMT: 21 NHE-DMT: 14 No DMT: 8
Calvi et al. (2022a)^3^	Retrospective analysis of RCT	MC	2015–2016	345	345 SPMS	55.6 ± 1.8^c^	230 F 115 M	19.8 ± 1.2^c^	Baseline, 24, 96	2	6 ± 0.2^c^	No DMT: 345
Calvi et al. (2022b)^27^	Retrospective observational cohort study	MC	Not specified	135	135 RRMS	35.5 ± 9	99 F 36 M	10.9 ± 6.2^c^	Variability of time intervals; baseline, ≥48	6.5 ± 2.3^c^	2.1 ± 1^c^	Type of DMT not specified at baseline: 66 Type of DMT not specified at the last follow-up: 102
Calvi et al. (2023)^8^	Retrospective observational cohort study	MC	Not specified	61	6 CIS 55 RRMS	37 ± 11^c^	42 F 19 M	4.4 ± 3.6^c^	Variability of time intervals; baseline, ≥33.6	3.9 ± 1.6^c^	1.9 ± 1^c^	Any DMT: 51
Elliott et al. (2023)^28^	Retrospective analysis of RCT	MC	2017–2021	41	41 RMS	38.3 ± 11.5^c^	27 F 14 M	5.5 ± 1.8^c^	Baseline, 24, 48, 72	1.5	2.5	HE-DMT: 15 NHE-DMT: 26
Federau et al. (2024)^29^	Retrospective observational cohort study	MC	Not specified	117	Not specified	46.5 ± 13.9^c^	81 F 36 M	Not specified	Not specified	Not specified	Not specified	Not specified
Arnold et al. (2024)^30^	Retrospective analysis of RCT	MC	2017–2018	261	228 RRMS 33 SPMS	42.4 ± 10.7^c^	181 F 80 M	Not specified	Baseline, 12, 16, 20, 24, 48, and 96	2	Not specified	HE-DMT: 207 NHE-DMT: 54
Yokote et al. (2024)^31^	Retrospective observational cohort study	MC	2016–2022	99	99 RRMS	40.3 ± 8.6^c^	74 F 25 M	9.6 ± 7.1^c^	Baseline, 24, 48, 72, 96	2 ± 0.2	2 ± 0.6^c^	HE-DMT: 42 NHE-DMT: 49
Huerta et al. (2024)^32^	Retrospective observational cohort study	SC	Not specified	15	15 RRMS	42.4 ± 5.6	12 F 3 M	8.5 ± 4.1	Baseline, 24, 48	1	2.7 ± 1.6^c^	HE-DMT: 15
Nakamura et al. (2024)^33^	Retrospective analysis of RCT	MC	2013–2017	195	107 PPMS 88 SPMS	55.9 ± 7	112 F 83 M	Not specified	Baseline, 24, 48, 72, 96	2	5.3 ± 1.5^c^	NHE-DMT: 97 Placebo: 98
Calvi et al. (2025)^34^	Prospective observational cohort study	SC	2011–2025	130	117 RRMS 7 PPMS 6 SPMS	41.8 ± 10.9^c^	93 F 37 M	7.7 ± 8^c^	Baseline, 25	8.13 ± 3.8^c^	1.5 ± 0.8^c^	Any DMT: 94 No DMT: 36

Abbreviations: CIS = clinically isolated syndrome; DMT = disease-modifying treatment; EDSS = Expanded Disability Status Scale; F = female; HE-DMT = high efficacy-disease-modifying treatment; M = male; MC = multicenter; MS = multiple sclerosis; NHE-DMT = non–high efficacy-disease-modifying treatment; PPMS = primary progressive multiple sclerosis; PRMS = progressive-relapsing multiple sclerosis; RCT = randomized controlled trial; RRMS = relapsing-remitting multiple sclerosis; SEL = slowly expanding lesion; SC = single center; SPMS = secondary progressive multiple sclerosis.

HE-DMT: Alemtuzumab, cladribine, evobrutinib, fingolimod, natalizumab, ocrelizumab, ofatumumab, opicinumab, ozanimod, rituximab, and siponimod.

NHE-DMT: Dimethyl fumarate, glatiramer acetate, interferon β-1a, interferon β-1b, peginterferon β-1a, and teriflunomide.

a Analyzed cohort for the presence of SELs.

b n = 554.

c Means and SDs were calculated using an automated tool.^13^

**Table 2:** MRI Scan Characteristics and Segmentation Methods

Study	Field strength	Scanner model (manufacturer)	Methodology of SEL identification	Sequence(s) for primary identification of SELs	Sequence(s) for lesion mask	Minimum voxels for identification of SELs	JE1	JE2	Concentricity	Constancy
Fox et al. (2016)^20^	1.5T	Siemens Avanto, Erlangen, Germany	Automated tool	T1w	Not specified	Not specified	Not specified	Not specified	Not specified	Not specified
Absinta et al. (2019)^21^	7T	Siemens Magnetom	Manually	T2* and phase contrast	T2*	CAGR ≥1%	Not specified	Not specified	Not specified	Not specified
Elliott et al. (2018)^22^	1.5T and 3T	Not specified	ANTs	T1w and T2w	T2	10	12.5%	4%	≥0	≥0
Elliott et al. (2020)^23^	1.5T and 3T	Not specified	ANTs	T1w and T2w	T2	10	12.5%	4%	≥0	≥0
Klistorner et al. (2020)^19^	3T	GE Discovery MR750	Automated in-house software package	FLAIR	FLAIR	50	10%	—	—	—
Klistorner et al. (2021)^12^	3T	GE Discovery MR750	Automated in-house software package	FLAIR	FLAIR	100	10%	—	—	—
Moog et al. (2022)^24^	3T	Philips Medical Systems, Cleveland, OH	In-house software package	T1w and T2w	FLAIR and T2w	3 mm^3^ and nonconfluent	Not specified	Not specified	Not specified	Centroid movement
Preziosa et al. (2022)^4^	3T	Intera, Philips Medical Systems, Best, The Netherlands	SPM12	T1w and T2w	T2	10	12.5% over 2 y	4%	≥0	≥0
Beynon et al. (2022)^25^	1.5T and 3T	Not specified	ANTs	T1w and T2w	T2	10 and not Gd+	12.5%	4%	≥0	≥0
Weber et al. (2022)^26^	3T	MAGNETOM Skyra, Siemens, Erlangen, Germany	I Automated in-house tool	T1w	FLAIR	5 mm in long axis	—	—	—	—
Calvi et al. (2022a)^3^	1.5T and 3T	Not specified	NiftyReg	T1w	FLAIR	10	12.5%	4%	≥0	≥0
Calvi et al. (2022b)^27^	1.5T and 3T	Gyroscan, Philips Healthcare, Best, The Netherlands, and Achieva, Philips, Eindhoven, The Netherlands	NiftyReg	T1w	FLAIR	10	12.5%	4%	≥0	≥0
Calvi et al. (2023)^8^	3T	Tim Trio; Siemens, Erlangen, Germany	NiftyReg	T1w	FLAIR	10	12.5%	4%	≥0	≥0
Elliott et al. (2023)^28^	3T	Not specified	ANTs	T1w and T2w	T2	10	12.5%	4%	≥0	≥0
Federau et al. (2024)^29^	1.5T and 3T	GE, Siemens, and Philips	Jazz	FLAIR						
Arnold et al. (2024)^30^	1.5T and 3T	Not specified	ANTs	T1w and T2w	T2	10	12.5%	4%	≥0	≥0
Yokote et al. (2024)^31^	1.5T and 3T	(T) Vantage Titan (Canon), MAGNETOM Spectra (Siemens), Discovery MR750w (GE Healthcare), and MAGNETOM Aera (Siemens)	ANTs	T1w	FLAIR	10	12%	8%	≥0	≥0
Huerta et al. (2024)^32^	7T	Siemans Magnetom (Siemens Healthineers)	SPM12	T1w	FLAIR	10	12.5%	4%	≥0	≥0
Nakamrura et al. (2024)^33^	3T	Siemens or GE	ANTs	T1w and T2w	T2	10	12.5%	4%	≥0	≥0
Calvi et al. (2025)^34^	3T	Siemens Magnetom Trio/Prisma	NiftyReg	T1w and T2w	FLAIR	10	12.5%	4%	≥0	≥0

Abbreviations: ANT = advanced normalization tool; CAGR = compound annual growth rate; Gd = gadolinium; GE = General Electric; FLAIR = fluid-attenuated inversion recovery; JE1 = Jacobian Expansion between baseline and time point 1; JE2 = Jacobian expansion between baseline and time point 2; SEL = slowly expanding lesion; SPM12 = Statistical Parametric Mapping software 12; T1w = T1 weighted; T2w = T2 weighted.

### Methodology of SEL and PRL Detection

**Figure 1:**
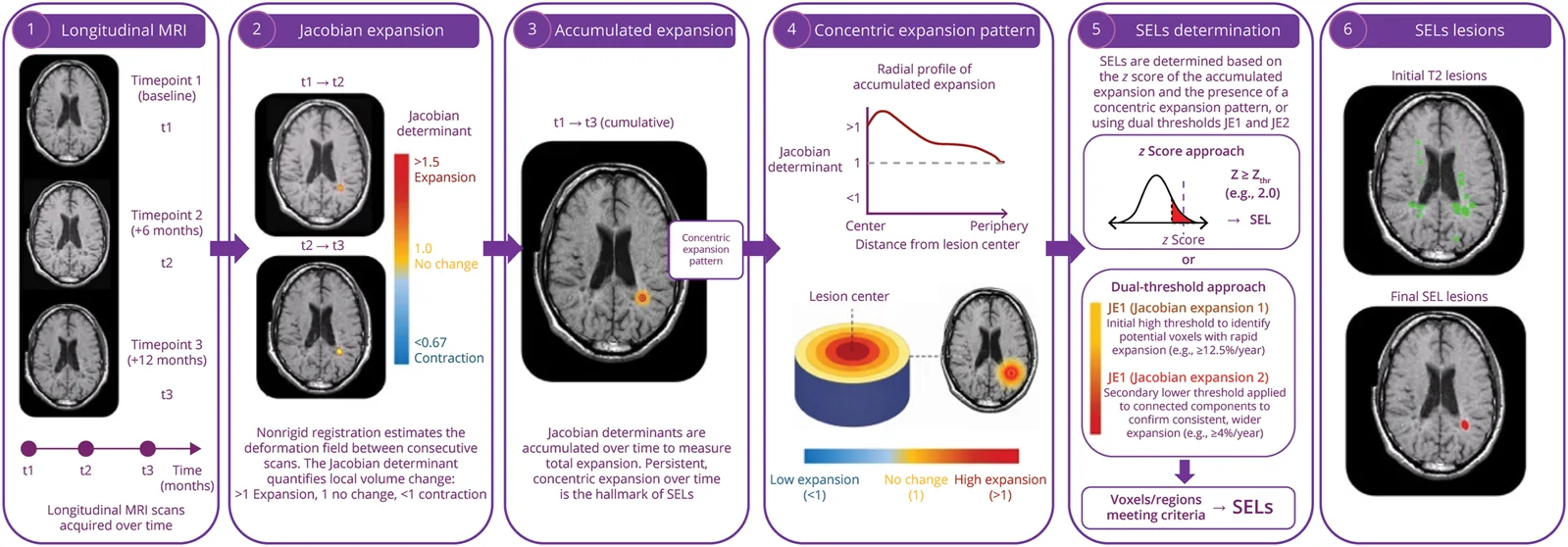
Schematic Overview of SEL Detection on Longitudinal MRI Schematic overview of the general pipeline for identifying SELs in MS using longitudinal MRI (1). Nonrigid registration of consecutive scans enables computation of Jacobian determinant maps to quantify local volume changes (2). Temporal accumulation of expansion (3) within preexisting lesions reveals a characteristic concentric pattern (4), reflecting chronic active inflammation. SELs are distinguished (5–6) from other T2 lesions and serve as imaging biomarkers of disease progression. This schema only summarizes and illustrates the most used approaches (*z*-score or dual threshold). MS = multiple sclerosis; SEL = slowly expanding lesion.

All studies, except 1,^21^ used automated tools to identify SELs. Most used Jacobian (deformation-based) methods (Figure 1),^36^ using various software packages, such as advanced normalization tools (ANTs), Statistical Parametric Mapping 12 (SPM12), or NiftyReg; 5 studies used in-house automated software, based on lesional volumetric change.^12,19,20,24,29^

In studies using the Jacobian analysis method, SELs were defined as lesions with concentric, gradual, linear expansion over time on serial T1-weighted and/or T2-weighted scans, measuring at least 10 voxels in size and exhibiting local expansion.^3,4,8,22,23,25,27,28,30-34^ In these studies, except 1,^31^ the minimum local expansion used to define voxels within the SELs mask was 4% per year. In 2 studies, lesion expansion was identified using a custom-designed algorithm, with a threshold of 50^19^ or 100 voxels^12^ (Table 2). For the identification of SELs, T1-weighted and/or T2-weighted images were used in all studies, except for 3 studies, in which SELs were identified on FLAIR images using an in-house tool.^12,19,29^

PRLs were identified using susceptibility-weighted imaging (SWI) and phase-based techniques, acquired at varying field strengths (e.g., 1.5T, 3T, and 7T).^8,21,28^ Across studies, PRL detection relied on expert visual assessment of the presence of a partial or complete hypointense rim at the edge of nonenhancing T2 lesions, confirmed on consecutive slices and across orthogonal planes, with exclusion of potential mimics (e.g., veins or susceptibility artefacts), although specific criteria varied across studies.

### Meta-Analysis

**Figure 2:**
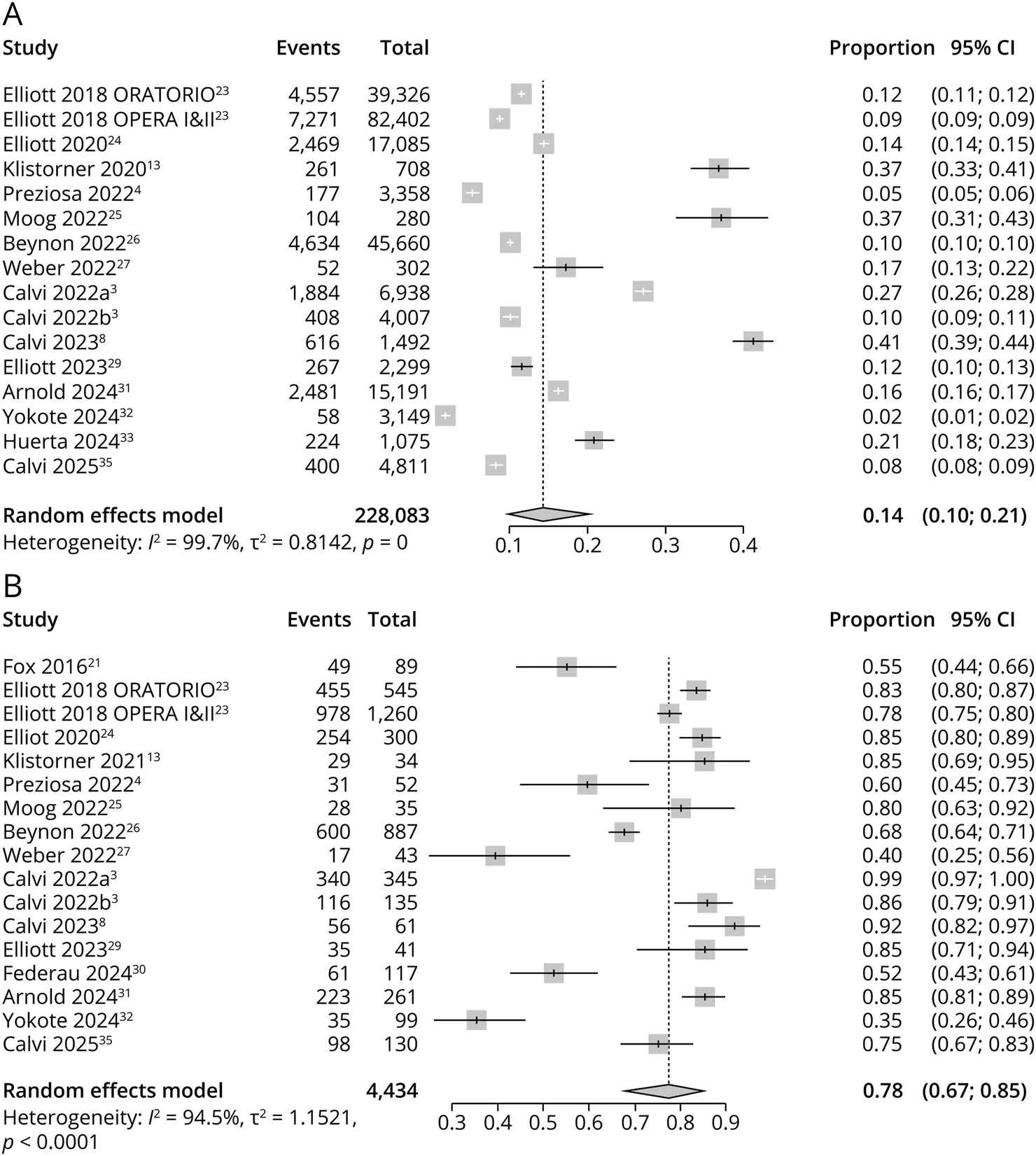
Proportion of SELs at Lesion and Patient Levels (A) Forest plot of study-specific and pooled estimates from a random-effects model showing the pooled proportion of SELs among all T2 lesions in people with MS. (B) Forest plot of study-specific and pooled estimates from a random-effects model showing the pooled proportion of people with MS with at least on SEL. MS = multiple sclerosis; SEL = slowly expanding lesion.

**Figure 3:**
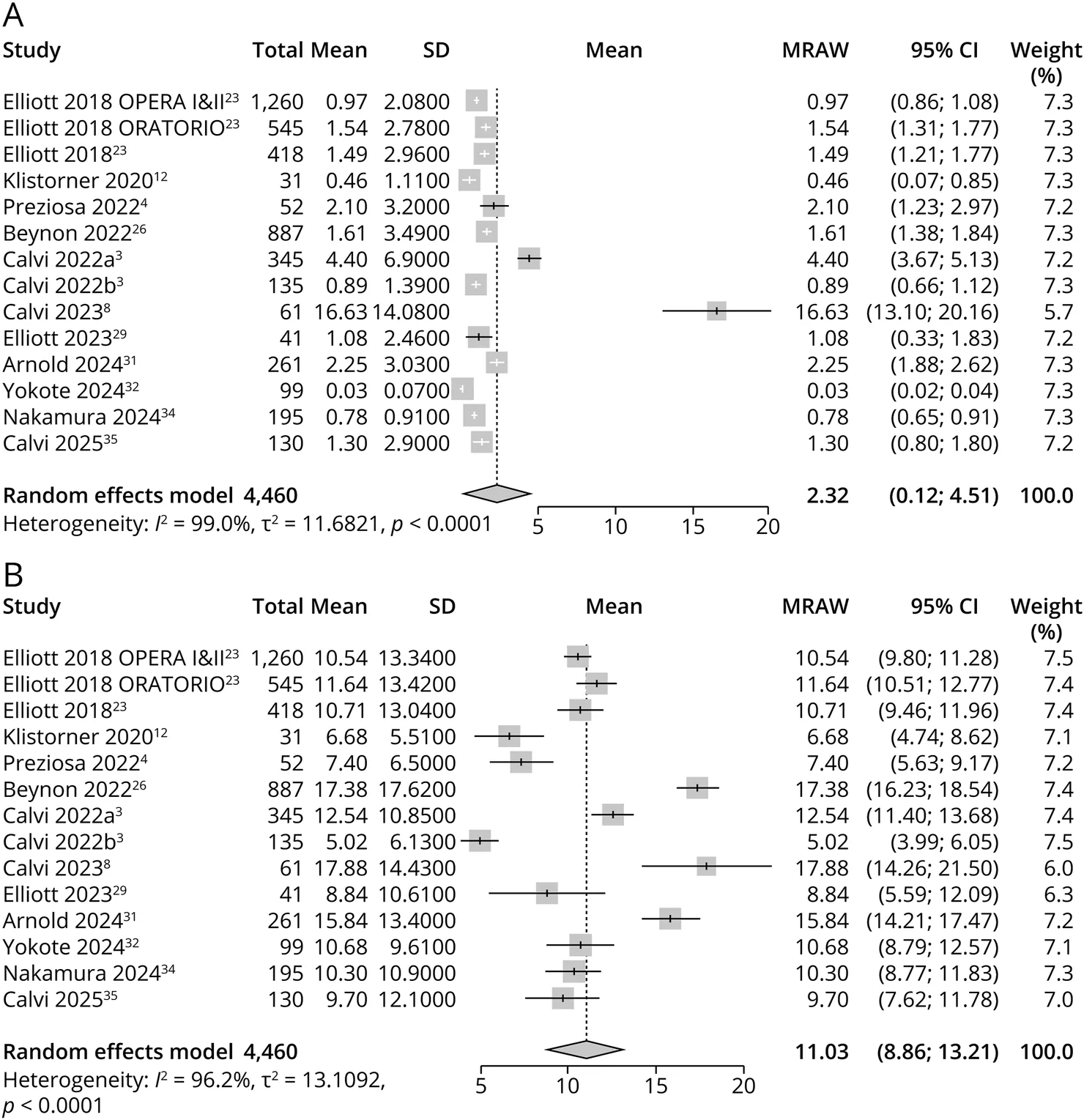
Mean Per-Patient Volume of SELs and Total T2 Lesions in People With MS (A) Forest plot of study-specific and pool estimates from a random-effects model showing the mean per-patient volume of SELs in people with MS. (B) Forest plot of study-specific and pool estimates from a random-effects model showing the mean per-patient volume of total T2 lesions in people with MS. MRAW = mean raw; MS = multiple sclerosis; SEL = slowly expanding lesion.

**Figure 4:**
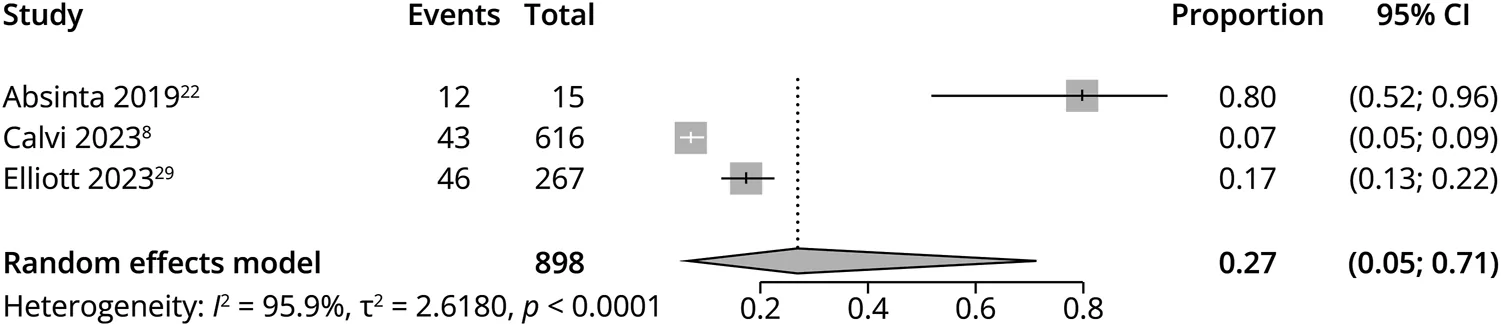
Proportion of SELs That Exhibit Characteristics of PRLs Forest plot of study-specific and pooled estimates from a random-effects model showing the pooled proportion of SELs that exhibit characteristics of PRLs in people with MS. MS = multiple sclerosis; PRLs = paramagnetic rim lesions; SEL = slowly expanding lesion.

Sixteen reports from 15 original studies, including 4,240 people with MS,^3,4,8,19,22-28,30-32,34^ were included in the lesion-level analysis, yielding a pooled SEL proportion (out of all T2 lesions) of 14% (95% CI 10%–21%) with high between-study heterogeneity (*I*^2^ = 99.7%) (Figure 2A). Seventeen reports from 16 original studies, including 4,434 people with MS,^3,4,8,12,20,22-31,34^ were included in the patient-level analysis, yielding a pooled proportion of patients with at least 1 SEL of 78% (67%–85%) with high between-study heterogeneity (*I*^2^ = 95.1%) (Figure 2B). Mean per-patient volumes, pooled from 14 studies with a total of 4,189 people with MS,^3,4,8,19,22,23,25,27,28,30,31,33,34^ were 2.32 mL (0.12–4.51) for SELs and 11.03 mL (8.86–13.21) for total T2 lesions, with high between-study heterogeneity (*I*^2^ = 99% and *I*^2^ = 99%, respectively) (Figure 3). To estimate the pooled proportion of SELs that were also identified as PRLs, we pooled 3 studies, including 125 people with MS.^8,21,28^ The pooled proportion was 27% (5%–71%), with high between-study heterogeneity (*I*^2^ = 95.9%) (Figure 4).

### Subgroup Analysis, Outlier Detection, Influence and Sensitivity Analysis, and Meta-Regression

To explore potential sources of heterogeneity, we compared RRMS with progressive MS (PMS; including both PPMS and SPMS). Although lesion-level prevalence was similar between the 2 groups, with high between-study heterogeneity (RRMS: 15% [7%–28%, *I*^2^ = 99.7%]; PMS: 15% [9%–24%, *I*^2^ = 99.9%]) (eFigure 2, eAppendix 9), the proportion of people with MS with at least 1 SEL was higher in PMS (87% [69%–95%, *I*^2^ = 96%])^3,4,22,23,25^ than RRMS (76% [65%–85%; *I*^2^ = 92.7%])^4,8,12,20,22-24,27,28,31^ (eFigure 3, eAppendix 9).

In Jacobian-only studies (Figure 1) (N = 4,174 people with MS,)^3,8,25,27,30,31,34^ lesion-level prevalence of SELs was 12% (8%–18%, *I*^2^ = 99.7%) (eFigure 4, eAppendix 9). In addition, when subgroup analyses were performed according to study design, separating RCTs and observational studies, the proportion of SELs out of all T2 lesions was comparable between RCTs and observational cohorts (14% [10%–18%] vs 15% [8%–27%], respectively) (eFigures 5 and 6, eAppendix 9). By contrast, patient-level analyses showed a higher proportion of individuals with at least 1 SEL in RCT-derived cohorts (86% [75%–93%]) compared with observational studies (69% [55%–80%]) (eFigures 7 and 8, eAppendix 9). Between-study heterogeneity remained substantial in all subgroup analyses. Outliers affected 2 analyses: removing Absinta et al.^21^ reduced pooled proportion of SELs identified as PRLs to 11% (6%–20%, *I*^2^ = 95.1%) (eFigure 9, eAppendix 9) and excluding Calvi et al.^8^ lowered pooled mean volumes to 1.42 mL (0.79–2.06, *I*^2^ = 99.1%) for SELs and 10.6 mL (8.53–12.67, *I*^2^ = 96%) for total T2 lesions (eFigure 10, eAppendix 9). However, heterogeneity remained high, indicating potential residual variability across the remaining studies.

Exploratory univariable meta-regression analyses did not identify significant associations between SEL prevalence and the examined study-level variables at either the lesion or patient level (Table 3). At both the lesion and patient levels, pooled proportion of SELs showed no significant correlation with the interval from baseline to final MRI (ρ = −0.01, *p* = 0.1 for both analyses) or with mean age (ρ = 0.04, *p* = 0.5; ρ = 0.08, *p* = 0.18). At the patient level, exploratory analyses suggested possible trends toward a higher proportion of SEL-positive patients among those not receiving DMTs (ρ = 19.4, *p* = 0.06) and a lower proportion among those receiving DMTs (ρ = −20.9, *p* = 0.05); however, these associations did not reach statistical significance.

**Table 3:** Meta-Regression Analysis of Study-Level Moderators at Lesion and Patient Levels

Variables	Lesion level			Patient level		
	Residual heterogeneity *I*^2^ (%)	Estimate	*p* Value	Residual heterogeneity *I*^2^ (%)	Estimate	*p* Value
Time frame of SEL identification	99.85	−0.01	0.10	99.85	−0.01	0.10
Mean age	99.88	0.04	0.40	96.76	0.08	0.18
Disease duration	99.88	−0.001	0.98	96.11	0.13	0.15
Baseline EDSS	99.86	−0.05	0.79	96.32	0.33	0.15
Percentage of patients received any DMT	99.88	0.02	0.90	96.49	−20.9	0.05
Percentage of patients received HE-DMT	99.92	−0.13	0.32	98.14	−10.46	0.38
Percentage of patients received NHE-DMT	99.82	0.14	0.70	95.3	5.27	0.66
Percentage of patients received no DMT	99.87	−2.13	0.85	96.62	19.14	0.06
Tesla strength of the scanner	99.90	7.0T: 0.80 3.0T: 0.66	0.30	97.70	−0.34	0.56
Proportion of RRMS	—	—	—	96.62	−1	0.16

Abbreviations: DMT = disease-modifying treatment; EDSS = Expanded Disability Status Scale; HE-DMT = high efficacy-disease-modifying treatment; NHE-DMT = non–high efficacy-disease-modifying treatment; RRMS = relapsing-remitting multiple sclerosis; SEL = slowly expanding lesion.

### Quality Assessment

Three studies were assessed as having low risk of bias, 16 as moderate risk of bias, and 1 as high risk of bias (eTable 1, eAppendix 9). The domains most frequently contributing to risk of bias involved sample representativeness and selection (Q1–Q3) and incomplete reporting of response rates (Q9).

## Discussion

This systematic review and meta-analysis show that SELs account for a minority of T2 lesions (14% [10%–21%]), but most patients (78% [67%–85%]) have at least 1 SEL, with mean per-patient volumes of 1.42 mL (0.79–2.06) for SELs and 10.6 mL (8.53–12.67) for total T2 lesions, after leave-one-out sensitivity analysis. The similar proportion of SELs out of all T2 lesions between RRMS and PMS (15% [7%–28%]), together with the higher proportion of patients with SELs in PMS than RRMS (87% [69%–95%] vs 76% [65%–85%]), suggests that SELs may be more widespread at the patient level in progressive MS, without clear evidence from these data of differences in lesion-level propensity among existing T2 lesions. Proportion of SELs out of all T2 lesions was similar in RCT-derived and observational cohorts. However, the higher proportion of patients with at least 1 SEL in RCTs (86% [75%–93%]) than in observational studies (69% [55%–80%]) suggests that cohort selection, imaging protocols, and longitudinal study design may influence patient-level prevalence estimates, rather than the difference necessarily reflecting underlying biology. The persistence of substantial heterogeneity across all subgroup analyses further indicates that study design alone does not account for the observed variability, highlighting the influence of methodological and population-related factors.

The partial overlap (11% [6%–20%]) between SELs and PRLs reinforces that these markers capture related but noninterchangeable aspects of chronic active inflammation. This is consistent with recent meta-analytic data showing that PRLs are present in approximately 12% of lesions and in about half of people with MS,^37^ indicating that only a subset of chronic active lesions exhibits paramagnetic rims. This observation may also be influenced by methodological differences in PRL identification across studies. PRLs were detected using susceptibility-sensitive MRI sequences, including SWI and phase-based techniques, acquired at varying field strengths (e.g., 1.5T, 3T, and 7T).^8,21,28^ In addition, PRL detection relied on visual assessment, with differences in criteria such as whether a partial or a complete hypointense rim was required and how confirmation across consecutive slices was implemented. Such variability may influence the sensitivity and specificity of PRL detection and, consequently, contribute to heterogeneity in the reported overlap estimates. SELs are defined by linear and concentric volumetric expansion over time, whereas CALs may follow more heterogeneous growth trajectories, and not all exhibit iron rims, as shown histopathologically.^38^ Moreover, although paramagnetic rims may persist for up to 7 years, longitudinal imaging studies suggest that PRLs typically expand only during the first 3.5 years before reaching a plateau in volume,^39,40^ consistent with their low-incidence, as reported in a recent meta-analysis.^37^ Lesions exhibiting both features are strongly associated with PIRA and future evolution to PIRA^22,41^ underscoring the complementary value of these imaging markers.

The meta-regression did not identify a statistically significant association between the estimated pooled proportion of SELs out of all T2 lesions and the length of the interval between baseline and the final MRI assessment used for SEL identification. Nevertheless, variability in imaging intervals and acquisition schedules across studies may plausibly influence SEL detection through both technical and biological mechanisms. From a technical perspective, longer intervals between scans may improve the robustness of Jacobian-based detection by reducing noise in volumetric measurements. From a biological perspective, lesion expansion is unlikely to proceed at a constant rate over time and may attenuate or stabilizes as lesions evolve,^36^ potentially reflecting changes in underlying inflammatory activity^36^ or the effects of treatment.^22,25,30,42^ In addition, differences in the duration of observation and the number of imaging time points may affect the ability to distinguish transient or acute lesion enlargement from sustained, low-grade expansion, with shorter intervals more likely to capture episodic inflammatory changes and longer observation periods favoring the identification of lesions with persistent expansion consistent with chronic active inflammation. These findings are accompanied by a nonsignificant negative trend between patient-level SEL prevalence and treatment exposure (ρ = −20.9, *p* = 0.05), which, however, should be interpreted cautiously and does not permit inference regarding treatment effects on chronic lesion activity.

Age is a critical determinant of disease trajectory in MS, influencing clinical progression, neurodegenerative processes, immune senescence, and therapeutic responsiveness.^43^ However, meta-regression analyses did not identify a statistically significant association between SEL prevalence and mean age nor did age explain between-study heterogeneity in SEL prevalence. SELs have also been reported in pediatric-onset MS, indicating that chronic lesion activity can begin early in the disease course.^44,45^ Together, these observations suggest that age alone is insufficient to explain the distribution of SELs across MS populations. Chronic lesion activity may begin early in the disease course, challenging the traditional view that compartmentalized inflammation is a feature exclusive to progressive MS.^46^ The widespread presence of SELs across all phenotypes further supports the concept of a disease continuum in which progressive pathologic mechanisms are active even in early stages. SELs were most frequently localized in periventricular and deep white matter, regions characteristically affected by focal demyelinating lesions.^47^ Although this spatial pattern is consistent with conventional lesion distribution in MS, the presence of SELs suggests an overlay of chronic, smoldering inflammation, influenced by compartmentalized inflammation, ineffective resolution of tissue injury, and CSF-mediated gradients of damage.^39^

Most studies used the Jacobian determinant to identify SELs, but implementation varied across tools (e.g., ANTs, SPM12, and NiftyReg). Variations in regularization strategies significantly affect SEL detection because each software applies distinct constraints on diffeomorphic transformations, making regularization choice critical for localizing volumetric differences.^48^ This issue is linked to the robustness of the registration method, the transformation regularity, and the numerical scheme for computing the Jacobian determinant.^49^ In nonlinear registration, deformation is determined by optimizing a similarity metric (i.e., normalized mutual information) between 2 images, so transformation quality is highly dependent on the chosen metric. Jacobian nonlinear transformation can precisely detect expanding lesions and estimate baseline volume but is confounded by intensity changes and boundary shifts, limiting true volumetric quantification.^22,36^ Manual delineation is more accurate but is time consuming and requires long follow-up because of slow lesion progression and variability in masking. Beyond these technical considerations, alternative longitudinal strategies have sought to quantify lesion expansion through direct volumetric comparison of binary lesion masks across consecutive time points.^12,19^ In such frameworks, scans are typically aligned to account for global brain atrophy, and lesion growth is defined as the net volumetric difference between masks rather than deformation-derived expansion. By relying on direct mask-based measurements, this approach may reduce spurious signals related to ventricular enlargement or lesion displacement over long follow-up periods, thereby reducing the risk of misclassifying displacement-related effects as true biological expansion. However, this increased specificity comes at the expense of feasibility because segmentation must be performed at each time point, limiting scalability in large cohorts. Thus, Jacobian-based approaches provide an approximation that can be modulated by different thresholds (e.g., Jacobian expansion 1 or 2) and z-scores (Figure 1), whereas another proposed method^19^ measures actual change, as derived from differences between consecutive binary lesion masks, with a set of logical rules determining the final lesion type. These differences highlight the need for standardized, reproducible frameworks that harmonize registration procedures, segmentation strategies, and expansion thresholds to improve comparability across studies.

Although most studies were judged to have low to moderate risk of bias, few were designed to estimate prevalence in population-representative samples. However, SEL identification was consistently performed using validated longitudinal MRI techniques, supporting strong internal validity.

The results of this meta-analysis should be interpreted in light of several limitations. Funnel plot asymmetry observed in our analyses suggests the possibility of publication bias (eFigures 11–13, eAppendix 9); however, this should be interpreted cautiously in the context of the substantial between-study heterogeneity. Funnel plot asymmetry may also reflect small-study effects, whereby smaller studies report more variable estimates, as well as genuine methodological and population differences across studies. In addition, the relatively small number of studies in some analyses and the use of proportional outcomes further limit the reliability of funnel plots for detecting publication bias. Accordingly, publication bias cannot be excluded and may have influenced the pooled estimates, particularly given the high heterogeneity observed across the proportional meta-analyses, as indicated by τ^2^ and *I*^2^, which remained largely unexplained despite exploratory subgroup and meta-regression analyses. Methodological heterogeneity in SEL identification represents another source of variability. Differences in MRI acquisition protocols, including the use of 2-dimensional (2D) vs 3-dimensional (3D) sequences, may further contribute to between-study heterogeneity. Where 2D sequences were used for initial lesion delineation, images were subsequently transformed into 3D space for longitudinal analysis; however, variability in acquisition characteristics may still have influenced lesion detection and contributed to between-study heterogeneity. Although most studies used Jacobian-based approaches, implementation varied across image registration frameworks (e.g., ANTs, NiftyReg, and SPM12), lesion-masking strategies, expansion thresholds, and reporting of segmentation parameters, potentially affecting the sensitivity and specificity of SEL detection. Variability in the timing and number of MRI time points for SEL identification further complicates comparability because differences in imaging intervals influence noise and robustness in Jacobian-based measurements and likely contributed to the observed heterogeneity. Population-level heterogeneity also warrants consideration. Many included studies were secondary analyses of RCTs; while methodologically rigorous, restrictive eligibility criteria may limit generalizability to real-world MS populations. In addition, the inclusion of studies spanning different publication years introduced variability in diagnostic criteria, disease-modifying therapy exposure, and MRI protocols. Incomplete reporting of data further limited the ability of meta-regression analyses to explain heterogeneity. Finally, despite comprehensive database searches, relevant studies, written in language other than English, indexed elsewhere, or unpublished data may have been missed.

In conclusion, SELs account for approximately 14% of MRI-detected lesions and, while representing a subset of the total lesion burden in MS, are present in 78% of people with MS. Their occurrence across all MS phenotypes indicates that chronic lesion activity is not restricted to progressive disease stages and is also present in RRMS. After sensitivity analysis, approximately 11% of SELs were also PRLs, suggesting that a subset of SELs reflects CAL pathology. Standardized detection methods and large, well-characterized observational cohorts are needed to enable more consistent prevalence estimates and to define the true population-level burden of SELs.

## Glossary

2D: 2-dimensional
3D: 3-dimensional
ANT: advanced normalization tool
CAL: chronic active lesion
CIS: clinically isolated syndrome
DMT: disease-modifying therapy
EDSS: Expanded Disability Status Scale
FLAIR: fluid-attenuated inversion recovery
MS: multiple sclerosis
PIRA: progression independent of relapse activity
PMS: progressive multiple sclerosis
PPMS: primary progressive multiple sclerosis
PRISMA: Preferred Reporting Items for Systematic Reviews and Meta-Analyses
PRL: paramagnetic rim lesion
PRMS: progressive-relapsing multiple sclerosis
RCT: randomized controlled trial
RRMS: relapsing-remitting multiple sclerosis
SEL: slowly expanding lesion
SPM12: Statistical Parametric Mapping 12
SPMS: secondary progressive multiple sclerosis
SWI: susceptibility-weighted imaging

## References

[1] Frischer JM, Weigand SD, Guo Y, et al. Clinical and pathological insights into the dynamic nature of the white matter multiple sclerosis plaque. Ann Neurol. 2015;78(5):710-721. doi:10.1002/ana.24497 26239536 PMC4623970

[2] Bagnato F, Sati P, Hemond CC, et al. Imaging chronic active lesions in multiple sclerosis: a consensus statement. Brain. 2024;147(9):2913-2933. doi:10.1093/brain/awae013 38226694 PMC11370808

[3] Calvi A, Carrasco FP, Tur C, et al. Association of slowly expanding lesions on MRI with disability in people with secondary progressive multiple sclerosis. Neurology. 2022;98(17):e1783-e1793. doi:10.1212/WNL.0000000000200144 35277438

[4] Preziosa P, Pagani E, Meani A, et al. Slowly expanding lesions predict 9-year multiple sclerosis disease progression. Neurol Neuroimmunol Neuroinflamm. 2022;9(2):e1139. doi:10.1212/NXI.0000000000001139 35105685 PMC8808355

[5] Müller J, Cagol A, Lorscheider J, et al. Harmonizing definitions for progression independent of relapse activity in multiple sclerosis: a systematic review. JAMA Neurol. 2023;80(11):1232-1245. doi:10.1001/jamaneurol.2023.3331 37782515

[6] Dal-Bianco A, Oh J, Sati P, et al. Chronic active lesions in multiple sclerosis: classification, terminology, and clinical significance. Ther Adv Neurol Disord. 2024;17:17562864241306684. doi:10.1177/17562864241306684 39711984 PMC11660293

[7] Jäckle K, Zeis T, Schaeren-Wiemers N, et al. Molecular signature of slowly expanding lesions in progressive multiple sclerosis. Brain. 2020;143(7):2073-2088. doi:10.1093/brain/awaa158 32577755

[8] Calvi A, Clarke MA, Prados F, et al. Relationship between paramagnetic rim lesions and slowly expanding lesions in multiple sclerosis. Mult Scler J. 2023;29(3):352-362. doi:10.1177/13524585221141964 PMC9972234 36515487

[9] Page MJ, McKenzie JE, Bossuyt PM, et al. The PRISMA 2020 statement: an updated guideline for reporting systematic reviews. BMJ. 2021;372:n71. doi:10.1136/bmj.n71 33782057 PMC8005924

[10] Stroup DF, Berlin JA, Morton SC, et al. Meta-analysis of observational studies in epidemiology: a proposal for reporting. JAMA. 2000;283(15):2008-2012. doi:10.1001/jama.283.15.2008 10789670

[11] Ouzzani M, Hammady H, Fedorowicz Z, et al. Rayyan: a web and mobile app for systematic reviews. Syst Rev. 2016;5(1):210. doi:10.1186/s13643-016-0384-4 27919275 PMC5139140

[12] Klistorner S, Barnett MH, Yiannikas C, et al. Expansion of chronic MS lesions is associated with an increase of radial diffusivity in periplaque white matter. Mult Scler J. 2021;28(5):697-706. doi:10.1177/13524585211033464 34378454

[13] Munn Z, Moola S, Lisy K, et al. Methodological guidance for systematic reviews of observational epidemiological studies reporting prevalence and incidence data. Int J Evid Based Healthc. 2015;13(3):147-153. doi:10.1097/xeb.0000000000000054 26317388

[14] Wan X, Wang W, Liu J, et al. Estimating the sample mean and standard deviation from the sample size, median, range and/or interquartile range. BMC Med Res Methodol. 2014;14(1):135. doi:10.1186/1471-2288-14-135 25524443 PMC4383202

[15] Balduzzi S, Rücker G, Schwarzer G. How to perform a meta-analysis with R: a practical tutorial. BMJ Mental Health. 2019;22(4):153-160. doi:10.1136/ebmental-2019-300117 PMC10231495 31563865

[16] Schwarzer G, Rücker G. Meta-analysis of proportions. In: Evangelou E, Veroniki AA, eds. Meta-Research: Methods and Protocols. Springer US; 2022:159-172. doi:10.1007/978-1-0716-1566-9_10 34550590

[17] Schwarzer G, Chemaitelly H, Abu-Raddad LJ, et al. Seriously misleading results using inverse of Freeman-Tukey double arcsine transformation in meta-analysis of single proportions. Res Synth Methods. 2019;10(3):476-483. doi:10.1002/jrsm.1348 30945438 PMC6767151

[18] Higgins JPT, Thompson SG. Quantifying heterogeneity in a meta-analysis. Stat Med. 2002;21(11):1539-1558. doi:10.1002/sim.1186 12111919

[19] Klistorner S, Barnett MH, Yiannikas C, et al. Expansion of chronic lesions is linked to disease progression in relapsing–remitting multiple sclerosis patients. Mult Scler J. 2021;27(10):1533-1542. doi:10.1177/1352458520974357 33215557

[20] Fox J, Kraemer M, Schormann T, et al. Individual assessment of brain tissue changes in MS and the effect of focal lesions on short-term focal atrophy development in MS: a voxel-guided morphometry study. Int J Mol Sci. 2016;17(4):489. doi:10.3390/ijms17040489 27043553 PMC4848945

[21] Absinta M, Sati P, Masuzzo F, et al. Association of chronic active multiple sclerosis lesions with disability in vivo. JAMA Neurol. 2019;76(12):1474-1483. doi:10.1001/jamaneurol.2019.2399 31403674 PMC6692692

[22] Elliott C, Wolinsky JS, Hauser SL, et al. Slowly expanding/evolving lesions as a magnetic resonance imaging marker of chronic active multiple sclerosis lesions. Mult Scler J. 2018;25(14):1915-1925. doi:10.1177/1352458518814117 PMC6876256 30566027

[23] Elliott C, Arnold DL, Chen H, et al. Patterning chronic active demyelination in slowly expanding/evolving white matter MS lesions. AJNR Am J Neuroradiol. 2020;41(9):1584. doi:10.3174/ajnr.A6742 32819894 PMC7583098

[24] Moog TM, McCreary M, Wilson A, et al. Direction and magnitude of displacement differ between slowly expanding and non-expanding multiple sclerosis lesions as compared to small vessel disease. J Neurol. 2022;269(8):4459-4468. doi:10.1007/s00415-022-11089-9 35380254

[25] Beynon V, George IC, Elliott C, et al. Chronic lesion activity and disability progression in secondary progressive multiple sclerosis. BMJ Neurol Open. 2022;4(1):e000240. doi:10.1136/bmjno-2021-000240 PMC9185385 35720980

[26] Weber CE, Wittayer M, Kraemer M, et al. Long-term dynamics of multiple sclerosis iron rim lesions. Mult Scler Relat Disord. 2022;57:103340. doi:10.1016/j.msard.2021.103340 35158450

[27] Calvi A, Tur C, Chard D, et al. Slowly expanding lesions relate to persisting black-holes and clinical outcomes in relapse-onset multiple sclerosis. Neuroimage Clin. 2022;35:103048. doi:10.1016/j.nicl.2022.103048 35598462 PMC9130104

[28] Elliott C, Rudko DA, Arnold DL, et al. Lesion-level correspondence and longitudinal properties of paramagnetic rim and slowly expanding lesions in multiple sclerosis. Mult Scler J. 2023;29(6):680-690. doi:10.1177/13524585231162262 PMC10176750 37036134

[29] Federau C, Hainc N, Edjlali M, et al. Evaluation of the quality and the productivity of neuroradiological reading of multiple sclerosis follow-up MRI scans using an intelligent automation software. Neuroradiology. 2024;66(3):361-369. doi:10.1007/s00234-024-03293-3 38265684 PMC10859335

[30] Arnold DL, Elliott C, Martin EC, et al. Effect of evobrutinib on slowly expanding lesion volume in relapsing multiple sclerosis. Neurology. 2024;102(5):e208058. doi:10.1212/WNL.0000000000208058 38335474 PMC11067693

[31] Yokote H, Miyazaki Y, Fujimori J, et al. Slowly expanding lesions are associated with disease activity and gray matter loss in relapse-onset multiple sclerosis. J Neuroimaging. 2024;34(6):758-765. doi:10.1111/jon.13243 39390692

[32] Huerta MM, Conway DS, Planchon SM, et al. Longitudinal myelin content measures of slowly expanding lesions using 7T MRI in multiple sclerosis. J Neuroimaging. 2024;34(4):451-458. doi:10.1111/jon.13209 38778455

[33] Nakamura K, Thoomukuntla B, Bena J, et al. Ibudilast reduces slowly enlarging lesions in progressive multiple sclerosis. Mult Scler J. 2024;30(3):369-380. doi:10.1177/13524585231224702 PMC11190892 38286755

[34] Calvi A, Vivó Pascual F, Solana E, et al. Diffusivity anisotropy signature of the slowly expanding lesions predicts progression independent of relapse activity in multiple sclerosis. J Neurol Neurosurg Psychiatry. 2026;97(5):405-412. doi:10.1136/jnnp-2025-335884 40897401 PMC13151513

[35] Haddaway NR, Page MJ, Pritchard CC, et al. PRISMA2020: an R package and Shiny app for producing PRISMA 2020-compliant flow diagrams, with interactivity for optimised digital transparency and Open Synthesis. Campbell Syst Rev. 2022;18(2):e1230. doi:10.1002/cl2.1230. 36911350 PMC8958186

[36] Nakamura K, Guizard N, Fonov VS, et al. Jacobian integration method increases the statistical power to measure gray matter atrophy in multiple sclerosis. Neuroimage Clin. 2014;4:10-17. doi:10.1016/j.nicl.2013.10.015 24266007 PMC3830061

[37] Tranfa M, Cantoni V, Green R, et al. Prevalence and incidence of paramagnetic rim lesions in multiple sclerosis: a systematic review and meta-analysis. Radiology. 2025;316(3):e243718. doi:10.1148/radiol.243718 40985834

[38] Popescu BF, Frischer JM, Webb SM, et al. Pathogenic implications of distinct patterns of iron and zinc in chronic MS lesions. Acta Neuropathol. 2017;134(1):45-64. doi:10.1007/s00401-017-1696-8 28332093 PMC5486634

[39] Dal-Bianco A, Grabner G, Kronnerwetter C, et al. Slow expansion of multiple sclerosis iron rim lesions: pathology and 7 T magnetic resonance imaging. Acta Neuropathol. 2017;133(1):25-42. doi:10.1007/s00401-016-1636-z 27796537 PMC5209400

[40] Dal-Bianco A, Grabner G, Kronnerwetter C, et al. Long-term evolution of multiple sclerosis iron rim lesions in 7 T MRI. Brain. 2021;144(3):833-847. doi:10.1093/brain/awaa436 33484118

[41] Cagol A, Benkert P, Melie-Garcia L, et al. Association of spinal cord atrophy and brain paramagnetic rim lesions with progression independent of relapse activity in people with MS. Neurology. 2024;102(1):e207768. doi:10.1212/WNL.0000000000207768 38165377 PMC10834139

[42] Calvi A, Mendelsohn Z, Hamed W, et al. Treatment reduces the incidence of newly appearing multiple sclerosis lesions evolving into chronic active, slowly expanding lesions: a retrospective analysis. Eur J Neurol. 2024;31(1):e16092. doi:10.1111/ene.16092 37823722 PMC11236028

[43] Filippi M, Preziosa P, Barkhof F, et al. The ageing central nervous system in multiple sclerosis: the imaging perspective. Brain. 2024;147(11):3665-3680. doi:10.1093/brain/awae251 39045667 PMC11531849

[44] Fadda G, Banwell B, Elliott C, et al. Slowly expanding lesions differentiate pediatric multiple sclerosis from myelin oligodendrocyte glycoprotein antibody disease. Ann Neurol. 2024;96(6):1086-1091. doi:10.1002/ana.27066 39243229

[45] Pozzilli V, Prados Carrasco F, Kim N, et al. Slowly expanding lesions in pediatric-onset multiple sclerosis. JAMA Neurol. 2025;82(11):1197. doi:10.1001/jamaneurol.2025.2619 40788653 PMC12340671

[46] Kuhlmann T, Moccia M, Coetzee T, et al. Multiple sclerosis progression: time for a new mechanism-driven framework. Lancet Neurol. 2023;22(1):78-88. doi:10.1016/S1474-4422(22)00289-7 36410373 PMC10463558

[47] Thompson AJ, Banwell BL, Barkhof F, et al. Diagnosis of multiple sclerosis: 2017 revisions of the McDonald criteria. Lancet Neurol. 2018;17(2):162-173. doi:10.1016/S1474-4422(17)30470-2 29275977

[48] Ashburner J, Ridgway GR. Symmetric diffeomorphic modeling of longitudinal structural MRI. Front Neurosci. 2012;6:197. doi:10.3389/fnins.2012.00197 23386806 PMC3564017

[49] Lorenzi M, Ayache N, Frisoni GB, et al. Alzheimer’s Disease Neuroimaging Initiative (ADNI). LCC-Demons: a robust and accurate symmetric diffeomorphic registration algorithm. Neuroimage. 2013;81:470-483. doi:10.1016/j.neuroimage.2013.04.114 23685032

